# Neurocognitive endophenotypes in neurodevelopmental disorders: social cognition and executive functions in children with Autism Spectrum Disorder Level 1 and Attention-Deficit/Hyperactivity Disorder

**DOI:** 10.3389/frcha.2026.1920753

**Published:** 2026-09-14

**Authors:** Grazia Maria Giovanna Pastorino, Miriam Olivieri, Antonio Aquino, Noemi Mungo, Rossella Suriano, Francesca Felicia Operto

**Affiliations:** 1 Department of Health Sciences, Magna Graecia University of Catanzaro, Catanzaro, Italy; 2 Departement of Medicine and Surgery, University of Salerno, Baronissi, Italy; 3 Department of Clinical and Experimental Medicine, University of Messina, Messina, Italy

**Keywords:** ADHD, affect recognition, Autism Spectrum Disorder Level 1, executive functions, social cognition, theory of mind

## Abstract

**Introduction:**

Executive Functions (EF) and Social Cognition (SC) show strong interdependence, yet it remains unclear whether social difficulties in neurodevelopmental disorders, such as Autism Spectrum Disorder Level 1 (ASD1) and Attention Deficit/Hyperactivity Disorder (ADHD), arise from shared EF related mechanisms or reflect disorder specific neurocognitive architectures. Comparative studies integrating both ecological and performance based measures remain scarce. This study aims to analyze the relationship between executive functions and Social Cognition skills across three matched developmental groups: Autism Spectrum Disorder Level 1 (ASD1), Attention-Deficit/Hyperactivity Disorder (ADHD), and Typical Development (TD).

**Methods:**

A total of 123 children aged 6–11 years participated in the study: ASD1 (*N* = 42), ADHD (*N* = 43), and TD (*N* = 38). Participants were evaluated using the WISC-IV, BRIEF-2, and selected subtests of the NEPSY-II. Non-parametric group comparisons and multiple linear regression models within the clinical groups were performed.

**Results:**

Both clinical groups showed widespread EF and SC impairments compared to TD peers, but with distinct profiles. ADHD exhibited pronounced deficits in inhibition and attentional control (Inhibition, Auditory/Visual Attention), whereas ASD1 showed greater impairment in SC (ToM, AR), cognitive flexibility (Shift), and rule-guided tasks (Response Set, DesignFluency). Regression models revealed disorder-specific EF–SC relationships: in ADHD, SC performance was strongly scaffolded by EF (Inhibition, Auditory Attention, Design Fluency; ToM model R^2^ = 0.72). In ASD1, ToM was predicted primarily by AR and Response Set (R^2^ = 0.55), indicating relative independence from basic inhibitory and attentional resources.

**Conclusions:**

Despite superficially similar social difficulties, ASD1 and ADHD display distinct patterns of association between EF and SC: executive-regulatory deficits in ADHD versus primary mentalizing and shifting impairments in ASD1. These findings support the need for transdiagnostic profiling to optimize early screening and to guide targeted, domain-specific rehabilitative and psychoeducational interventions for each diagnostic profile.

## 1 Introduction

Social cognition (SC) is a core neurocognitive domain that allows individuals to process, interpret, and adaptively respond to socially relevant cues, thereby supporting effective interpersonal functioning and communication (1–3). Within SC, Affect Recognition (AR) provides the perceptual basis for higher-order socio-cognitive abilities (4–7), including Theory of Mind (ToM)- the capacity to attribute mental states such as beliefs, intentions, and emotions to oneself and others to guide flexible social behavior (1, 8).

ToM is a multidimensional construct comprising cognitive, affective, and intermediate components. These components are supported by partially distinct neural systems, including the medial prefrontal cortex, temporoparietal junction, superior temporal sulcus, and limbic structures (9).

In parallel, Executive Functions (EF) constitute a set of top-down regulatory processes—response inhibition, working memory, planning, cognitive flexibility, and attentional control—primarily mediated by prefrontal and frontostriatal circuits (10, 11). EF regulate goal-directed behavior and self-control and are essential for adaptive functioning across development (12, 13).

Dysfunctions in EF and SC represent transdiagnostic vulnerabilities across childhood-onset Neurodevelopmental Disorders, particularly Autism Spectrum Disorder (ASD) and Attention-Deficit/Hyperactivity Disorder (ADHD) (14–16). While inhibitory control deficits are generally more prominent in ADHD, ASD is more frequently associated with impairments in cognitive flexibility (or set-shifting, defined here as the higher-order executive ability to adapt cognitive strategies and change mental rules) and planning, although substantial overlap exists across executive domains (17–21). Separately, deficits in basic attentional control—including sustained attention and attentional shifting (the lower-order ability to orient the attentional focus between different sensory stimuli without altering underlying cognitive rules)—have also been consistently documented in ASD, suggesting that attentional dysfunction extends beyond social processing and may contribute to broader executive inefficiency (22–25). Social difficulties are also shared but tend to be more severe and specific in ASD (1).

Historically, neuropsychological models proposed a distinctive pattern of EF: ADHD was characterized by primary deficits in inhibition and working memory (10), while ASD was associated with impairments in flexibility and planning (12, 26). However, recent comparative studies challenge this view. Meta-analytic evidence shows that both groups display comparable impairments in direct tasks assessing inhibition, flexibility, and attention (27–29). Conversely, ecological questionnaires consistently reveal more severe everyday executive difficulties in ADHD, particularly in inhibition and organization (30–32).

In contrast, Social Cognition profiles diverge more clearly. ToM deficits in ASD are considered primary and pervasive, affecting false-belief reasoning, perspective taking, and social perception (33–35). Children with ADHD may show reduced performance on cognitive ToM tasks, but these difficulties are largely attributable to attentional fluctuations and inhibitory demands embedded in the tasks (1, 6). When controlling for executive load, ToM performance in ADHD often approaches typical levels (36).

Clarifying the directionality of the EF-SC relationship is therefore crucial. Developmental models propose that EF provide the cognitive scaffolding necessary for ToM (37–39), but this relationship may differ across neurodevelopmental profiles (36, 40). Evidence remains mixed, partly due to methodological heterogeneity and the limited number of studies directly comparing ASD and ADHD using standardized measures across both domains.

This gap is particularly relevant in middle childhood, a developmental window characterized by rapid and asynchronous maturation of executive control and social reasoning (41–43). Clarifying shared and distinct neurocognitive mechanisms during this period may support more accurate transdiagnostic profiling and guide early, individualized interventions.

The present study addresses these gaps by conducting a comparative analysis of EF and SC in three matched groups of children with ASD Level 1, ADHD and typically developing peers, using both direct neuropsychological tasks and indirect ecological measures (3, 36). A further objective was to examine the predictive relationship between EF and SC within each clinical group to determine whether the EF–ToM link follows similar or distinct developmental pathways in ASD and ADHD. By integrating standardized assessments across domains and focusing on a critical developmental stage, this study aims to contribute to a more nuanced understanding of transdiagnostic and disorder-specific neurocognitive profiles, with implications for early screening and personalized intervention planning (14, 16, 32).

## 2 Methods

### 2.1 Study design and ethical considerations

This multi-center, cross-sectional, comparative observational study was conducted between 2021 and 2025 in two distinct public healthcare centres in Italy: the Child and Adolescent Neuropsychiatry Day Hospital at the University Hospital of Salerno, and the Child and Adolescent Neuropsychiatry Outpatient Clinic at the University Hospital Dulbecco of Catanzaro.

The study was conducted in accordance with the ethical principles of the Declaration of Helsinkiand was approved by the Ethics Committee “Campania Sud”.

Parents/caregivers received detailed information about the study's methods and objectives and provided written informed consent. All collected data were anonymized and processed in agreement with current European and national privacy regulations (GDPR).

### 2.2 Sample selection

The clinical sample included children aged 6–11 years with a formal diagnosis of Autism Spectrum Disorder—Level 1—(ASD1) or Attention-Deficit/Hyperactivity Disorder—combined presentation (ADHD), established according to the Diagnostic and Statistical Manual of Mental Disorders, Fifth Edition (DSM-5) criteria and strictly validated by a public health Child and Adolescent Neuropsychiatrist. Diagnostic validation employed gold-standard tools: the Autism Diagnostic Observation Schedule, Second Edition (ADOS-2) and the Autism Diagnostic Interview-Revised (ADI-R) were administered for the ASD1 group, while the Conners 3rd Edition (Conners-3) parent/teacher rating scales were employed for the ADHD group. We collected socio-demographic (age, sex, Socio-Economic Status—SES) and clinical information for all the participants. SES was determined based on parental educational attainment and professional occupation, and subsequently categorized into three distinct operational levels (1 = Low, 2 = Medium, 3 = High).

Inclusion criteria were: (i) age between 6 and 11 years; (ii) formal diagnosis of either ASD1 or ADHD (combined presentation); and (iii) good compliance of the participants and their families to complete performance-based neuropsychological testing and to fill out behavioral questionnaires.

Exclusion criteria for all participants were: (i) the presence of a comorbid neurodevelopmental diagnosis (specifically, children with both ASD and ADHD diagnoses were strictly excluded to ensure the study of ‘pure' clinical phenotypes); (ii) an Intelligence Quotient (IQ) below 70 (to exclude comorbid Intellectual Disability); and (iii) the presence of any major neurological, psychiatric, or systemic medical conditions that could interfere with the neuropsychological and behavioral evaluations.

Specifically, a Child Neuropsychiatrist excluded major medical and neurological conditions (such as cerebral palsy, epilepsy, movement disorders, migraine, metabolic diseases, chronic internal pathologies, and known genetic syndromes) through clinical assessment and instrumental examinations. Simultaneously, the multidisciplinary team ensured the exclusion of neurodevelopmental and psychiatric comorbidities (including anxiety or mood disorders, psychosis, and tic disorders, as well as the presence of dual ASD and ADHD diagnoses) using comprehensive clinical interviews and standardized diagnostic tools, based on DSM-5 criteria. Methylphenidate therapy in the ADHD group was not an exclusion criterion, however participants were asked not to take it before the neuropsychological tests.

Typically developing (TD) controls were recruited from a healthy pediatric population participating in a school-based screening program for Specific Learning Disorders (SLDs). Children who screened positive for SLDs during the program were excluded. TD participants met the same inclusion/exclusion criteria as clinical groups and were matched for age, sex, and socioeconomic status (SES).

### 2.3 Measures and procedures

Neuropsychological assessments were completed in two separate sessions conducted by specialized personnel (three developmental neuropsychologists with almost 10 years of experience). To ensure optimal testing conditions, the children were evaluated individually in a quiet, dedicated room designed to minimize external environmental stimuli and distractions. Concurrently, parent-report questionnaires were administered to the parents in a separate, dedicated room. The performance-based testing required approximately 90–120 min of active engagement from the children, while the parental questionnaires took approximately 30 min to complete.

#### 2.3.1 Wechsler intelligence scale for children—fourth edition (WISC-IV)

The WISC-IV, standardized and validated for the Italian population, was used to assess cognitive functioning. This test provides four Index scores: Verbal Comprehension Index (VCI), Perceptual Reasoning Index (PRI), Working Memory Index (WMI), and Processing Speed Index (PSI), which collectively determine the Full-Scale Intelligence Quotient (FSIQ). Raw scores were converted into age-normed standardized index scores with a normative mean of 100 and a standard deviation (SD) of 15. Higher scores indicate superior cognitive functioning. The WISC-IV possesses robust psychometric properties, demonstrating excellent internal consistency (alpha >.90 for core indices), high test-retest reliability and strong construct validity.

#### 2.3.2 Behavior rating inventory of executive function—second edition (BRIEF-2)

Parents completed the BRIEF-2 Parent Form to evaluate the everyday behavioral manifestations of executive functions in the home environment. We refer to the BRIEF-2 as an ecological assessment tool, as it captures the child's functioning in real-world settings as perceived by caregivers; however, it remains an informant-report rating scale rather than a direct, objective performance-based measure. The scale includes nine clinical domains aggregated into the Global Executive Composite (GEC): Inhibit, Self-Monitoring, Shift, Emotional Control, Initiate, Working Memory, Plan/Organize, Task-Monitor, and Organization of Materials. Raw scores were converted into age- and sex-standardized T-scores (mean = 50, SD = 10). Higher T-scores indicate a greater severity of executive dysfunction, with scores > 65 indicating clinically significant impairment. The BRIEF-2 has demonstrated exceptional internal consistency (alpha = .90 to.97 for composite scales), strong test-retest stability, and high discriminative validity in differentiating neurodevelopmental disorders (such as ASD1 and ADHD) from typically developing populations.

#### 2.3.3 A developmental neuropsychological assessment—second edition (NEPSY-II)

We assessed Social Cognition and Executive Functions using selected subtests from the NEPSY-II, a comprehensive developmental neuropsychological battery. Based on the study's aims, subtests were drawn from two primary domains:

1. Social Cognition: Affect Recognition (AR), which assesses the ability to recognize emotional expressions from photographs of children's faces; and Theory of Mind (TOM), which measures the capacity to understand others' mental states, beliefs, and perspectives.

Attention and Executive Functions: Inhibition, evaluating the ability to suppress automatic responses; Visual Attention that assesses selective attention; Auditory Attention and Response Set subtest. These components were scored separately to differentiate basic attentional control from higher-order executive functions: the Auditory Attention component assesses basic sustained and selective auditory attention, whereas the Response Set component evaluates cognitive flexibility (set-shifting), inhibition of automatic responses, and the maintenance of complex, conflicting auditory rules; and Design Fluency, which evaluates non-verbal productivity and cognitive flexibility (set-shifting). Raw scores for each subtest were transformed into age-standardized scores with a mean of 10 and an SD of 3. Higher scores reflect better performance. The NEPSY-II shows high validity, adequate to strong internal consistency (reliability coefficients ranging from.70 to.90) and robust test-retest reliability across clinical groups.

### 2.4 Statistical analysis

Data are expressed as percentage, mean as standard deviation. Preliminarily, the normality of the distribution for continuous variables was assessed using the Shapiro–Wilk test. Given the deviation from the normality assumption found in most of the clinical and behavioral measures (particularly within the BRIEF-2 and NEPSY-II scales; *p* < .05), a non-parametric approach was adopted. Baseline homogeneity among the three groups (ASD1, ADHD, TD) regarding continuous sociodemographic variables (age) was verified using the Kruskal–Wallis test, whereas the Chi-square test was employed for categorical differences (Sex, SES).

To investigate differences among the diagnostic groups in cognitive (WISC-IV), behavioral (BRIEF-2), and executive-social (NEPSY-II) profiles, the Kruskal–Wallis test for independent samples was employed. Wherever a significant result emerged, *post-hoc* comparisons were performed using the Mann–Whitney U test. To control the error deriving from multiple comparisons, *p*-values were adjusted by applying the Bonferroni correction (*p* = .05/3; *p* < .0167). To evaluate the magnitude of the effects, effect sizes for these pairwise contrasts were calculated using the rank-biserial correlation coefficient (r), where values of.10,.30, and.50 represent small, medium, and large effects, respectively.

Finally, to examine the impact of executive functions on social cognition skills within the two clinical groups (ASD1 and ADHD), multiple linear regression models were implemented. For all analyses, the statistical significance level was considered *p* = .05 (two-tailed). Statistical analyses were conducted using SPSS software, version 25.

## 3 Results

### 3.1 Participants

The final sample consisted of 123 children: Autism Spectrum Disorder—Level 1 (ASD1) group (*N* = 42), Attention-Deficit/Hyperactivity Disorder (ADHD) group (*N* = 43), and Typically Developing (TD) control group (*N* = 38). Demographic, clinical and socioeconomic characteristics for each group are summarized in Table 1. The global results of statistical analysis showed that the three groups did not significant differ for age, sex, and socioeconomic status (Table 1).

**Table 1:** Sociodemographic characteristics of the sample.

Variables	ASD1 (*n* = 42)	ADHD (*n* = 43)	TD (*n* = 38)	Statistical analysis
Age	8.88 ± 1.47 (mean ± SD)	9.09 ± 1.48 (mean ± SD)	9.16 ± 1.42 (mean ± SD)	Kruskal–Wallis H *p* = 0.648
Sex (male)	28/42 (66.7%)	27/43 (62.8%)	23/38 (60.5%)	Chi-Square test *p* = 0.846
SES Low Medium High	10 (23.8%) 24 (57.1%) 8 (19.1%)	5 (11.6%) 31 (72.1%) 7 (16.3%)	9 (23.7%) 20 (52.6%) 9 (23.7%)	chi square = 4.087 *p* = 0.394
Pharmacotherapy		Methylphenidate *n* = 12/43 (28%) mean dose = 8.13 mg/die		

SD, standard deviation; ASD1, Autism Spectrum Disorder—Level 1; ADHD, Attention-Deficit/Hyperactivity Disorder; TD, typically developing; SES, socio-economic status.

### 3.2 Neuropsychological and executive profiles: comparative patterns across groups

To highlighted the main differences between ASD1, ADHD and TD, we synthesized the salient results of comparative analysis by weaknesses of each group (summarized in Table 2).

- **Domains of greater impairment in ASD1:** Children with ASD1 exhibited a significantly more severe clinical presentation compared to the ADHD group in social cognition tasks (Theory of Mind and Affect Recognition of Nepsy-II) and in cognitive flexibility/set shifting abilities, both in performance test (Response Set and Design Fluency of Nepsy-II) and in everyday life (Shift Scale of BRIEF-2).
- **Domains of greater impairment in ADHD:** Conversely, the ADHD group displayed significantly more profound deficits compared to the ASD1 group in top-down regulatory control, specifically regarding response inhibition and basic attentional processes, both in everyday life (BRIEF-2 Inhibit scale) and during direct performance-based tasks (Inhibition, Visual Attention, and Auditory Attention subtests).
- **Domains of shared neurodevelopmental vulnerability:** Both clinical groups exhibited widespread and comparable executive dysfunction relative to TD controls in working memory capacity (WMI subscale of WISC-IV) and overall everyday executive functioning (Global Executive Composite of BRIEF-2), with no significant differences found between the two clinical conditions.
- **Domains with no reliable group differences:** All three groups remained strictly homogeneous with respect to core fluid intelligence, verbal processing, and speed of processing indices, showing no significant variations in Verbal Comprehension (VCI), Perceptual Reasoning (PRI), and Processing Speed (PSI) of WISC-IV.

**Table 2:** Synthesis of comparative analysis of neuropsychological and executive profiles between ASD1, ADHD and TD.

Comparative Interpretive Pattern	Cognitive/Behavioral Domain	Standardized Measure	Post-Hoc Contrast Direction	Statistics
**Greater impairment in ASD1** *(Both clinical groups are impaired vs. TD, but ASD1 shows the most severe deficit)*	-Theory of Mind - Affect Recognition - Everyday flexibility/shifting - Cognitive flexibility/set shifting	-Nepsy-II, ToM - Nepsy-II, AR - BRIEF-2, Shift - Nepsy-II, Response Set/Design Fluency	ASD < ADHD < TD	***p*** **<** **0.05**
**Greater impairment in ADHD** *(Both clinical groups are impaired vs. TD, but ADHD shows the most severe deficit)*	-Everyday inhibition - Response Inhibition - Basic attentional control - Everyday global executive functioning	- BRIEF-2, Inhibit - Nepsy-II, Inhibition - Nepsy-II, Visual Attention/Auditory Attention - BRIEF-2, GEC	ADHD < ASD1 < TD	***p*** **<** **0.05**
**Shared impairment vs. TD** *(No significant difference between ASD1 and ADHD)*	- Working Memory	- WISC-IV, WMI	ASD = ADHD < TD	***p*** **<** **0.05**
**No group differences**	-Verbal Comprehension -Perceptual Reasoning -Processing Speed	-WISC-IV, VCI -WISC-IV, PRI -WISC-IV, PSI	ASD1 = ADHD = TD	*p* > 0.05

ASD1, autism spectrum disorder level 1; ADHD, attention deficit/hyperactivity disorder; TD, tipical developing. the direction of the *post-hoc* contrasts is reported based on subject performance, where a lower position indicates greater impairment, regardless of whether the specific tool uses direct scores (WISC-IV, NEPSY-II) or inverted scores (BRIEF-2). Significant *p*-value are in bold.

Detailed, test-by-test metrics for each neurocognitive and behavioral domain are reported in the subsequent subsections and corresponding tables.

#### 3.2.1 Cognitive profile (WISC-IV)

.Comparative analyses revealed that fluid intelligence and general verbal abilities remained highly homogeneous across the three cohorts, as no significant differences emerged for the Verbal Comprehension Index (VCI), Perceptual Reasoning Index (PRI), and Processing Speed Index (PSI) (*p* > 0.05).

In contrast, a highly significant group difference was observed for the Working Memory Index (WMI) (*p* < 0.001, H = 15.95). *Post-hoc* analyses with Bonferroni correction confirmed a pattern of shared vulnerability: both the ASD1 and the ADHD groups performed significantly lower than the TD group, with medium-large effect sizes (ASD1 vs. TD: **r** **=** .387; ADHD vs. TD: r = .493), while no significant performance discrepancy was found between the two clinical cohorts (*p* = .949). Finally, a significant difference was observed on Full-Scale IQ (FSIQ) (*p* = .042, H = 6.35), driven exclusively by a relative reduction in the ADHD group compared to TD controls (*p* = .031, r = .336). Findings are summarized in Table 3.

**Table 3:** Cognitive profile (WISC-IV) comparisons among groups.

WISC-IV	Mean ± SD ASD1	Mean ± SD ADHD	Mean ± SD TD	Statistical analysis	Post-Hoc Test
VCI	95.66 ± 18.15	96.12 ± 12.19	100.97 ± 11.93	*p* = .163 H = 3.63	-
PRI	102.02 ± 16.82	98.44 ± 12.52	99.13 ± 13.18	*p* = .648 H = 3.63	-
WMI	81.80 ± 12.75	80.51 ± 1010	90.39 ± 11.15	*p* < .001 H = 15.95	ASD1 vs. TD (*p* = .009, **r** **=** **.387**), ADHD vs. TD (*p*=<.001, **r** **=** **.493**))
PSI	92.93 ± 12.65	91.37 ± 10.97	91.24 ± 10.45	*p* = .671 H = 0.80	-
FSIQ	92.07 ± 16.44	90.07 ± 10.99	96.92 ± 8.92	*p* = .042 H = 6.35	ADHD vs. TD (*p* = .031, **r** **=** **.336**)

SD, standard deviation; ASD1, Autism Spectrum Disorder—Level 1; ADHD, Attention-Deficit/Hyperactivity Disorder; TD, typically developing; VCI, verbal comprehension index; PRI, perceptual reasoning index; WMI, working memory index; PSI, processing speed index; FSIQ, full-scale IQ. Data are presented as Mean ± Standard Deviation. Comparisons were performed using the Kruskal–Wallis H test followed by Bonferroni-corrected Mann–Whitney U tests. Effect sizes are reported as rank-biserial correlation coefficient (r), where values of.10,.30, and.50 represent small, medium, and large effects, respectively (in bold).

#### 3.2.2 Behavioral executive profile (BRIEF-2)

In the everyday life contest, both clinical groups exhibited widespread executive dysfunction, scoring significantly worse across all BRIEF-2 subscales compared to TD controls (*p* < .001). However, the direct comparison between the two neurodevelopmental conditions revealed a significant difference between the two groups.

For the Global Executive Composite (GEC), the ADHD group showed a significantly higher score compared to the ASD1 group (post-hoc *p* = .002, r = .428). Specifically, the ADHD group displayed the most severe executive impairment on the Inhibit scale, scoring significantly worse than the ASD1 group (*p* < .001, r = .500), corroborating the dominance of behavioral disinhibition in this profile. Conversely, children with ASD1 exhibited a significantly greater impairment on the Shift scale compared to the ADHD group (*p* = .024, r = .333), reflecting more pronounced everyday cognitive inflexibility, resistance to change, and perseverative behaviors. Results are summarized in Table 4.

**Table 4:** Executive function profiles (BRIEF-2) comparisons among groups.

BRIEF-2	Mean ± SD ASD1	Mean ± SD ADHD	Mean ± SD TD	Statistical analysis	Post-Hoc Test
Inhibit	60.83 ± 9.10	69.60 ± 8.72	41.50 ± 5.49	*p* < .001 H = 80.89	ASD1 vs. ADHD (*p*=<.001, **r** **=** **.500**), ASD1 vs. TD (*p*=<.001, **r** **=** **.930**), ADHD vs. TD (*p*=<.001, **r** **=** **.992**)
Self-Monitoring	62.10 ± 9.28	64.86 ± 8.07	44.37 ± 1.32	*p* < .001 H = 54.69	ASD1 vs. TD (*p*=<.001, **r** **=** **.784**), ADHD vs. TD (*p*=<.001, **r** **=** **.876**)
Shift	65.93 ± 9.92	60.37 ± 7.94	44.74 ± 6.62	*p* < .001 H = 65.39	ASD1 vs. ADHD (*p* = .024, **r** **=** **.333**), ASD1 vs. TD (*p*=<.001, **r** **=** **.914**), ADHD vs. TD (*p*=<.001, **r** **=** **.868**)
Emotional Control	62.40 ± 7.94	67.19 ± 8.09	43.50 ± 6.59	*p* < .001 H = 71.79	ASD1 vs. ADHD (*p* = .028, **r** **=** **.328**), ASD1 vs. TD (*p*=<.001, **r** **=** **.910**), ADHD vs. TD (*p*=<.001, **r** **=** **.959**)
Initiate	60.62 ± 8.94	68.88 ± 8.17	45.84 ± 7.58	*p* < .001 H = 68.31	ASD1 vs. ADHD (*p*=<.001, **r** **=** **.484**), ASD1 vs. TD (*p*=<.001,**r** **=** **.785**), ADHD vs. TD (*p*=<.001, **r** **=** **.956**)
Working Memory	63.43 ± 8.97	67.58 ± 9.12	42.53 ± 6.28	*p* < .001 H = 72.45	ASD1 vs. TD (*p*=<.001, **r** **=** **.940**), ADHD vs. TD (*p*=<.001, **r** **=** **.954**)
Plan/Organize	61.98 ± 9.59	66.31 ± 9.15	42.26 ± 6.80	*p* < .001 H = 68.74	ASD1 vs. TD (*p*=<.001, **r** **=** **.888**), ADHD vs. TD (*p*=<.001, **r** **=** **.961**)
Task-Monitor	61.10 ± 8.42	64.28 ± 8.36	44.84 ± 7.38	*p* < .001 H = 62.28	ASD1 vs. TD (*p*=<.001,**r** **=** **.862**), ADHD vs. TD (*p*=<.001, **r** **=** **.905**)
Organization of Materials	59.00 ± 9.27	66.44 ± 8.74	44.18 ± 6.64	*p* < .001 H = 67.17	ASD1 vs. ADHD (*p* = .005, **r** **=** **.389**), ASD1 vs. TD (*p*=<.001, **r** **=** **.791**), ADHD vs. TD (*p*=<.001, **r** **=** **.969**)
Global Executive Composite (GEC)	65.26 ± 7.25	71.30 ± 7.76	41.42 ± 7.67	*p* < .001 H = 81.95	ASD1 vs. ADHD (*p* = .002, **r** **=** **.428**), ASD1 vs. TD (*p*=<.001, **r** **=** **.986**), ADHD vs. TD (*p*=<.001, **r** **=** **.990**)

SD, standard deviation; ASD1, Autism Spectrum Disorder—Level 1; ADHD, Attention-Deficit/Hyperactivity Disorder; TD, typically developing. Data are presented as Mean ± Standard Deviation. Comparisons were performed using the Kruskal–Wallis H test followed by Bonferroni-corrected Mann–Whitney U tests. Effect sizes are reported as rank-biserial correlation coefficient (r), where values of.10,.30, and.50 represent small, medium, and large effects, respectively (in bold).

#### 3.2.3 Performance-based neuropsychological profile (NEPSY-II)

Direct performance-based testing supported and deepened the behavioral findings. While both clinical groups performed significantly lower than TD controls across all tasks (*p* < .001), the NEPSY-II subtests highlighted highly distinctive neurocognitive profiles characterized by different patterns of relative impairment.

The ASD1 group demonstrated the most profound comparative impairment within the Social Cognition domain. For Theory of Mind (ToM), the ASD1 group obtained a significantly lower score (mean = 5.33) than the ADHD group (mean = 6.93; *post-hoc p* = .004, r = .397). Similarly, the ASD1 group showed lower performance in Affect Recognition (AR) compared to the ADHD cohort (post-hoc *p* = .004, r = .405). Regarding executive tasks, the ASD1 group performed significantly worse than the ADHD group in Response Set (*p* = .008, d = r = .374) and Design Fluency (*p* = .024, r = .329), indicating specific difficulties in higher-order cognitive flexibility (set-shifting) and rule-guided strategy generation.

Conversely, the ADHD group exhibited the lowest scores in response inhibition and basic attentional tasks. In the Inhibition subtest, the ADHD group performed significantly worse than the ASD1 group (post-hoc *p* = .004, r = .396). For Visual Attention the ADHD group performing worse than the ASD1 group (*p* = .044, r  =  .304). The same trend was confirmed for Auditory Attention, where the ADHD group was significantly more impaired than the ASD1 cohort (*p* = .048, r = .299).

All results are summarized in Table 5.

**Table 5:** Social cognition and executive functions tasks (NEPSY-II) comparison among groups.

NEPSY-II	Mean ± SD ASD1	Mean ± SD ADHD	Mean ± SD TD	Statistical analysis	Post-Hoc Test
*Social Cognition*					
AffectRecognition (AR)	4.67 ± 2.27	6.51 ± 2.42	10.42 ± 1.72	*p* < .001 H = 65.88	ASD1 vs. ADHD (*p* = .004, **r** **=** **.405**), ASD1 vs. TD (*p*=<.001,**r** **=** **.952**), ADHD vs. TD (*p*=<.001,**r** **=** **.762**)
Theory of Mind (TOM)	5.33 ± 1.78	6.93 ± 2.25	10.61 ± 1.57	*p* < .001 H = 69.75	ASD1 vs. ADHD (*p* = .004, **r** **=** **.397**), ASD1 vs. TD (*p*=<.001,**r** **=** **.981**), ADHD vs. TD (*p*=<.001,**r** **=** **.795**)
*Executive Functions*					
Inhibition	6.79 ± 1.96	5.30 ± 1.93	10.16 ± 1.81	*p* < .001 H = 65.27	ASD1 vs. ADHD (*p* = .004, **r** **=** **.396**), ASD1 vs. TD (*p*=<.001,**r** **=** **.773**), ADHD vs. TD (*p*=<.001,**r** **=** **.942**)
AuditoryAttention	6.24 ± 1.32	5.30 ± 1.86	10.08 ± 1.46	*p* < .001 H = 75.98	ASD1 vs. ADHD (*p* = .048,**r** **=** **.299**), ASD1 vs. TD (*p*=<.001,**r** **=** **.953**), ADHD vs. TD (*p*=<.001, **r** **=** **.967**)
Response Set	5.07 ± 1.89	6.28 ± 1.44	10.08 ± 1.71	*p* < .001 H = 75.75	ASD1 vs. ADHD (*p* = .008,**r** **=** **.374**), ASD1 vs. TD (*p* = <.001, **r** **=** **.958**), ADHD vs. TD (*p* = <.001, **r** **=** **.907**)
Design Fluency	5.38 ± 1.51	6.33 ± 1.49	9.89 ± 1.45	*p* < .001 H = 77.40	ASD1 vs. ADHD (*p* = .024,**r** **=** **.329**), ASD1 vs. TD (*p* = <.001, **r** **=** **1.000**), ADHD vs. TD (*p* = <.001, **r** **=** **1.000**)
Visual Attention	6.50 ± 1.70	5.64 ± 1.81	10.00 ± 1.36	*p* < .001 H = 69.00	ASD1 vs. ADHD (*p* = .044, **r** **=** **.304**), ASD1 vs. TD (*p* = <.001,**r** **=** **1.000**), ADHD vs. TD (*p* = <.001,**r** **=** **1.000**)

SD, standard deviation; ASD1, Autism Spectrum Disorder—Level 1; ADHD, Attention-Deficit/Hyperactivity Disorder; TD, typically developing. Data are presented as Mean ± Standard Deviation. Comparisons were performed using the Kruskal–Wallis H test followed by Bonferroni-corrected Mann–Whitney U tests. Effect sizes are reported as rank-biserial correlation coefficient (r), where values of.10,.30, and.50 represent small, medium, and large effects, respectively (in bold).

### 3.3 Relationship between social cognition and executive function in ADHD and ASD1

To investigate the complex interplay between executive functions and social-cognitive performance, a series of multiple linear regression models were constructed. Affect Recognition (AR) and Theory of Mind (ToM) were selected as the primary dependent variables, with performance-based executive function (EF) subtests modeled as predictors. Additionally, each social-cognitive measure was included as a predictor for the other. This modeling architecture is theoretically justified by dominant developmental neuropsychological frameworks, which state that efficient social cognition is a complex, top-down capacity that requires the functional integrity of cognitive functions (e.g., holding social cues in working memory, inhibiting irrelevant information, and shifting between perspectives). Furthermore, including the social-cognitive variables as mutual predictors allows for the exploration of the hierarchical dependencies within the socio-cognitive abilities, assessing whether affect recognition (AR) act as necessary prerequisites for conceptual mentalizing capacities (ToM), or vice versa, across distinct clinical profiles. All the results are summarized in Table 6.

**Table 6:** Multiple linear regression models predicting social cognition from executive functions within ASD1 and ADHD groups.

Clinical Group	DependentVariable	SignificantPredictors	Beta (β)	t	p
**ASD1**	AffectRecognition (AR)	Theory of Mind (TOM)	.583	3.20	.003
		Visual Attention	.370	2.15	.039
	Theory of Mind (TOM)	AffectRecognition (AR)	.389	3.20	.003
		Response Set	.514	3.95	<.001
**ADHD**	AffectRecognition (AR)	Inhibition	.462	2.48	.011
		Visual Attention	.327	2.09	.043
	Theory of Mind (TOM)	Inhibition	.312	2.21	.033
		AuditoryAttention	.338	3.33	.002
		Design Fluency	.266	3.03	.004

ASD1, Autism Spectrum Disorder—Level 1; ADHD, Attention-Deficit/Hyperactivity Disorder.

#### 3.3.1 Multiple regression models within the ASD1 group

The regression model for Affect Recognition was statistically significant, explaining 32.7% of the variance [adjusted R2 = .327, F(6,35) = 4.32, *p* = .002]. Within this model, ToM was a significant positive predictor (ß = .583, t = 3.20, *p* = .003); Visual Attention subtest also emerged as a moderate positive predictor (ß = .370, t = 2.15, *p* = .039) whereas the other variables included in the model did not show statistically significant effects in this sample.

For Theory of Mind, the model explained 55.1% of the variance [adjusted R2 = .551, F(6,35) = 9.38, *p* < .001], with Affect Recognition (ß = .389, t = 3.20, *p* = .003) and Response Set scores (ß = .514, t = 3.95, *p* < .001) acting as significant positive predictors.

#### 3.3.2 Multiple regression models within the ADHD group

The model for Affect Recognition explained 60.7% of the variance [adjusted R2 = .607, F(6,36) = 11.80, *p* < .001], primarily driven by Inhibition (ß = .462, t = 2.48, *p* = .011) and by Visual Attention (ß = .327, t = 2.09, *p* = .043). Unlike the ASD1 group, ToM did not predict Affect Recognition performance.

Regarding Theory of Mind, the model accounted for 72.2% of the variance [adjusted R2 = .722, F(6,36) = 19.17, *p* < .001], with Inhibition (ß = .312, t = 2.21, *p* = .033), Auditory Attention (ß = .338, t = 3.33, *p* = .002), and Design Fluency (ß = .266, t = 3.03, *p* = .004) serving as the main executive drivers.

## 4 Discussion

The present study investigated Executive Functions (EF) and Social Cognition (SC) in school-aged children with Autism Spectrum Disorder Level 1 (ASD1), Attention-Deficit/Hyperactivity Disorder (ADHD), and typically developing peers, using a multimodal assessment integrating ecological and performance-based measures. Consistent with transdiagnostic models of neurodevelopmental disorders (14, 44), both clinical groups showed widespread impairments across EF and SC domains. However, the pattern of results revealed distinct neurocognitive architectures, supporting the hypothesis that superficially similar social difficulties arise from different underlying mechanisms in ASD1 and ADHD.

### 4.1 Cognitive profile: shared and distinct vulnerabilities

The two clinical groups showed comparable performance on Verbal Comprehension (VCI), Perceptual Reasoning (PRI), and Processing Speed (PSI), confirming that group differences were not attributable to global cognitive impairment. In contrast, both ASD1 and ADHD groups showed significantly reduced Working Memory Index (WMI), confirming working memory as a shared endophenotype across neurodevelopmental conditions (10, 38). Regarding the shared impairment observed in the Working Memory Index (WMI) across both clinical groups, our data confirm a comparable phenotypic reduction but do not allow for a direct investigation of the underlying cognitive drivers, as this fell beyond the original objectives of the study. However, based on the existing literature, we hypothesize that working memory deficits may be underpinned by distinct, profile-specific vulnerabilities. Specifically, models in neurodevelopmental research suggest that the WMI reduction in ADHD may be primarily secondary to attention difficulties and interference-control failures (45). Conversely, in ASD1, it may be modulated by rigid encoding strategies or reduced strategic monitoring during information retention (13). Ultimately, these pathways remain tentative hypotheses that require direct regression and mediation modeling in future investigationsThe slight reduction in FSIQ observed only in ADHD aligns with evidence that attentional dysregulation and impulsivity can depress global cognitive performance without indicating true intellectual impairment (46). On the other hand, the performance within the normal limits in the two clinical groups in PRI confirmed that fluid, non-verbal Intelligence was preserved in both clinical groups.

### 4.2 Executive profile in everyday functioning

Both clinical groups showed significantly higher clinical severity indices at BRIEF-2 than controls. However, the data suggest a differential profile of everyday executive functioning. Children with ADHD showed the most severe global executive dysfunction and significantly greater impairment on the Inhibit scale compared to the ASD1 group, consistent with literature describing inhibitory dysregulation as a core feature of ADHD (30, 32). Conversely, children with ASD1 showed significantly higher impairment in Shift scalecompared to ADHD group, reflecting everyday cognitive inflexibility, resistance to change, and perseverative behaviours—hallmarks of executive dysfunction in autism (14, 31). These results suggest, in line with previous literature, a different clinical presentation of executive dysfunction in the two clinical groups: ADHD is dominated by behavioral disinhibition, whereas ASD1 is characterized by cognitive inflexibility and rule-bound behavior (24, 25, 47).

### 4.3 Performance-based EF and SC: evidence for disorder-specific neurocognitive architectures

Data derived from direct testing (NEPSY-II) corroborated and deepened the ecological findings. Children with ASD1 showed the most profound impairments in Theory of Mind (ToM) and Affect Recognition (AR). This pattern confirms that in autism without intellectual disability, the deficit in mentalization and decoding of social stimuli is primary, pervasive and not entirely explained by the generic executive load (8, 33). Their significantly reduced performance in Response Set and Design Fluency tasks suggests difficulties in cognitive flexibility (set shifting) and spontaneous strategy generation, aligning with models of executive rigidity in ASD (38, 48–50).

In contrast, children with ADHD showed the lowest performance in Inhibition, Visual Attention, and Auditory Attention, confirming the centrality of deficits in basic attentional control and response inhibition within ADHD neuropsychology (51–53). These findings support the notion that ADHD is characterized by a structural deficit in top-down regulatory control mediated by frontostriatal circuits.

### 4.4 EF–SC relationships: distinct developmental pathways in ASD1 and ADHD

Regression models suggest distinctt EF–SC architectures across the two clinical groups. In ASD1, the predictive model for Affect Recognition (AR) explained 32.7% of the variance, identifying ToM as its main positive predictor, complemented by a moderate contribution of Visual Attention. This strong interconnection suggests that in ASD1 the ability to decode a facial expression (AR, bottom-up process) is intimately linked to the abstract ability to attribute mental and emotional states (ToM, top-down process). These findings are consistent with developmental models proposing atypical integration of social perceptual and social cognitive systems (34, 54).

Equally informative is the model for ToM (55.1% of variance explained), where the only significant predictors were AR and Response Set. These data suggest that in ASD1, success in complex mentalization tasks requires both basic perceptual-social competence (AR) and higher-order executive skills related to cognitive flexibility (set-shifting) and rule monitoring (Response Set). Notably, basic attentional control and inhibitory functions do not exert a direct predictive role on ToM in ASD1, confirming the partial independence of the autistic socio-cognitive core from these lower-order executive components (8, 55). Our results confirm the primary and pervasive nature of socioemotional mentalization and decoding difficulties in ASD1, in line with our previous studies that have documented significant impairments in socio-relational skills and emotion processing in children with ASD1 (2).

In the ADHD group, the architecture of the relationships between SC and EF appears different. In the predictive model for AR (60.7% variance), ToM disappears as a predictor, while Visual Inhibition and Attention emerge as the only significant drivers. This indicates that the reduced performance of children with ADHD in recognizing facial emotions does not derive from an intrinsic deficit in social decoding, but rather from an impulsive and disorganized visual exploration of the stimulus, driven by deficits in basic attentional control and response inhibition. This interpretation is consistent with studies showing that when executive demands are minimized, ToM performance in ADHD approaches typical levels (6, 36).

Finally, the regression model for ToM in ADHD achieved a high explanatory power (72.2% of variance explained), being predicted by three executive variables: Inhibition, Auditory Attention, and Design Fluency. This result suggests that the Theory of Mind difficulties found in children with ADHD do not reflect a deficit of “pure” social competence (as in autism) but rather represent a direct consequence of the executive load inherent in the task. To solve a ToM task successfully, the child with ADHD must be able to keep attention focused on the narrative (basic attentional control via Auditory Attention), inhibit premature responses or distractions (response inhibition), and flexibly plan and generate possible cognitive solutions (higher-order cognitive flexibility via Design Fluency).

### 4.5 Strenghts and limitations

The present study has significant methodological strengths that support its scientific soundness. These include the multicenter design with consecutive recruitment, the rigorous selection of participants carried out through gold-standard tools and the careful matching of cohorts by age, sex and socioeconomic level. In addition, the decision to adopt a multimodal assessment protocol—direct and indirect standardized neuropsychological tests—has made it possible to overcome the intrinsic limits of individual diagnostic approaches, offering a multidimensional characterization of clinical profiles.

The study has several limitations: firstly, the study design is cross-sectional and does not allow to establish direct causal relationships or certain evolutionary trajectories in the long term; the age group considered (6–11 years) represents a period of asynchronous and rapid neuropsychological maturation, therefore, the architecture of the relationships found could undergo remodeling during adolescent transition.

Furthermore, a precise methodological choice concerned the exclusion of subjects with clinical comorbidities. In daily clinical practice, the co-occurrence of ASD and ADHD is frequent. Although the exclusion of comorbidity has been fundamental to isolate the cognitive profiles between ASD1 and ADHD, this criterion partially reduces the ecological validity and generalizability of the results to the heterogeneous clinical population that normally pertains to health services.

Moreover, the reliance on the BRIEF-2 as an ecological assessment introduces potential subjectivity. As an informant-report scale, these ratings may be influenced by parental perception, family stress, diagnostic expectations, and the specific context of the home environment. Unlike direct neuropsychological testing, these reports may not fully disentangle behavioral difficulties from the parent-child interaction dynamics, representing a limitation in our ability to isolate ‘pure' cognitive impairment from subjective report biases.

Finally, although multicenter recruitment has extended over a five-year period (2021–2025), the origin of the sample is limited to two specific public health realities in southern Italy. An expansion of the recruitment network at national or international level would make it possible to control any hidden cross-cultural or socio-environmental variables. Furthermore, the exclusive focus on Level 1 Autism (without intellectual disability) and on the combined presentation of ADHD does not allow these conclusions to be extended to nuanced phenotypic variants (such as ADHD with predominantly inattentional presentation) or to autism spectrum pictures with greater impairment of global functioning.

### 4.6 Clinical implications and future directions

With a view to overcoming these limitations, future research should prioritize the implementation of longitudinal study designs. A further development of crucial importance consists in the inclusion of a fourth experimental group composed of subjects diagnosed in comorbidity (ASD1 + ADHD). The neuropsychological analysis of this group would allow us to understand whether the co-occurrence of the two disorders generates an additive impairment profile (sum of ADHD inhibition deficits and ASD flexibility deficits), synergistic (exponential aggravation of one domain over the other) or whether it configures a distinct neurodevelopmental endophenotype.

From a technological and instrumental point of view, the integration of advanced eye-tracking and functional neuroimaging methods (such as fNIRS or quantitative fMRI) could strongly corroborate our behavioral models. Specifically, implementing eye-tracking protocols explicitly coupled with social-cognitive tasks would allow for the objective, real-time quantification of visual gaze patterns during Affect Recognition and Theory of Mind testing. This approach could clarify whether the distinct neuropsychological profiles identified in our study are underpinned by micro-behavioral visual strategies, such as a diminished fixation on the ocular region in ASD1 or chaotic, accelerated scanning paths driven by impulsivity in ADHD. Consequently, this would provide deeper mechanistic insights to design highly customized, gaze-contingent rehabilitation programs.

Regarding clinical utility, the results that emerged could give useful suggestions for the structuring of specific early screening protocols in the 6–11 age group, identified as a critical developmental window for executive functions and social cognition. These findings are also consistent with previous evidence showing that combined neuropsychological batteries can contribute to the differential characterization of ASD and ADHD profiles (56). At the same time, the study could offer useful indications for the planning of targeted and personalized intervention protocols, emphasizing that social difficulties cannot be treated in a standardized way, tailored to the real developmental needs of the child.

## 5 Conclusion

The present study helps to shed light on the complex phenotypic overlap that characterizes Autism Spectrum Disorder Level 1 (ASD1) and Attention-Deficit/Hyperactivity Disorder (ADHD) in the domains of Executive Functions of Social Cognition.

The results empirically suggest that the ASD1 profile shows a greater impairment in Social Cognition (Theory of Mind and Affect Recognition) than the ADHD profile and is also characterized by greater cognitive rigidity (difficulty in Shifting). On the other hand, the ADHD profile presents greater difficulties in Executive Functions, particularly in response inhibition and in both visual and auditory sustained attention processes.

Analysis of the relationship between Executive Functions and Social Cognition suggested that difficulties in ToM and emotion recognition are predicted by underlying executive and attentional difficulties. On the contrary, in ASD1 the socio-cognitive core is configured as a domain more independent of basic executive resources, requiring rather flexibility skills in executing complex rules.

In conclusion, these results underscore the transdiagnostic nature of some cognitive dysfunctions across different neurodevelopmental conditions, highlighting the critical necessity of enriching categorical diagnostic models with comprehensive, fine-grained neuropsychological profiling.. While our findings reveal distinct, profile-specific configurations unique to ASD1 and ADHD, the presence of shared vulnerabilities—such as working memory impairments -demonstrates that core cognitive deficits cut across traditional diagnostic boundaries. Therefore, these data suggest that categorical frameworks should not be viewed as rigid, isolated boxes; instead, they must be integrated with dimension-based cognitive mapping. This cross-disorder approach is essential to capture both the shared transdiagnostic features and the specific cognitive architectures of each patient, ultimately guiding the development of more targeted and flexible early screening protocols and personalized habilitative interventions,

## Data Availability

The raw data supporting the conclusions of this article will be made available by the authors, without undue reservation.
